# Phytochemicals reduced growth, sporulation and conidial dimensions of *Fusarium verticillioides*, cause of fumonisin contamination in maize grains

**DOI:** 10.1016/j.btre.2023.e00819

**Published:** 2023-10-30

**Authors:** Habtamu Terefe, Getnet Yitayih, Getachew G. Mengesha

**Affiliations:** a School of Plant Sciences, Haramaya University, P.O. Box 138, Dire Dawa, Ethiopia; b Department of Plant Sciences, Debre Tabor University, Debre Tabor, Ethiopia; c Arba Minch Agricultural Research Center, SARI, P.O. Box 2228, Arba Minch, Ethiopia

**Keywords:** Antifungal, Extracts, Fungal growth, *Fusarium*, Methanol

## Abstract

The objective of the study was to evaluate the effectiveness of methanolic extracts of plants against radial growth and spore dimensions of *Fusarium verticillioides*. Leaf extracts of 25 plants were tested against the fungus. Of which, thirteen extracts were potent against the fungus and evaluated using food poising technique. Growth was evaluated on PDA medium amended with the extracts at 5 mg ml^–1^. Control treatments included plates without (negative) extracts and with synthetic (positive) fungicide. Spore dimension was determined using PDB. The results showed *T. vulgaris* extract completely inhibited mycelial growth of the fungus as equivalent as the fungicide. Similarly, *G. parviflora, C. citratus, R. officinalis, R. chalepensis*, and *Agave* sp. also recorded growth reductions ranging from 71.04 to 81.35 % at day seven. In addition, extracts of *Agave* sp., *C. citratus* and *T. vulgaris* did not support sporulation. Overall, the results indicate that *T. vulgaris* extracts could be safe source of bioactive chemicals to control *F. verticillioides*.

## Highlights

• *Fusarium verticillioides* is responsible for fumonisin contamination in maize grains.
• Chemical based mold control is blamed for increased costs, handling hazards, residues and health risks.
• Phytochemicals are alternative management options for inhibiting toxigenic fungal growth.
• Phytochemicals of different origin reduced growth, sporulation and conidial dimensions of *F. verticillioides*.

## 1 Introduction

Cereals are the major food crops both in terms of the area they are planted and volume produced, when compared to other crops in Ethiopia. These crops contributed to 87.48 % of the total grain production. Maize, *teff*, wheat and sorghum made up 27.43, 17.26, 15.17 and 16.89 % of the grain production in the country, respectively [1]. However, several fungi genera constrain productivity of maize, sorghum, and wheat worldwide. Of which, in the past decades, there has been an increasing interest towards *Aspergillus, Penicillium, Fusarium*, and *Alternaria* general of fungi [2]. The genera *Aspergillus, Fusarium* and *Penicillium* were identified from maize grain samples collected from stores and markets from Ethiopia. Out of which, *F. verticillioides* was among the dominant toxigenic fungal species in the country [3], [4], [5] and other areas [6], [7], [8].

The occurrence of these fungi in foods and feeds may lead to significant economic losses, and health hazards to both humans and animals. This is mainly due to the production of mycotoxins [2]. To date, more than 300 mycotoxins have been identified [9,10]. The most commonly reported and characterized mycotoxins from maize and sorghum are mainly fumonisins and aflatoxins, which are associated with *F. verticillioides* and *A. flavus*, respectively, under the grain production systems of Ethiopia [3,11]. Many researchers reported that the occurrence of aflatoxins and fumonisins in food stuffs is highly associated with health risks to humans and animals [2,9,[12], [13], [14], [15], [16], [17]].

Most developing countries like Ethiopia are found in the tropics, where temperatures and relative humidity favor mold growth and subsequent aflatoxin and fumonisin contamination in food grains. The presence of mycotoxins is related to storage, environmental and ecological conditions of foods. Therefore, mycotoxin contamination can occur at various stages in the food chain [18,19]. The situation becomes much worsened to subsistence farmers in Ethiopia, where they used to harvest and dry grains in the field, transport and store them in underground pits with very poor sanitation that further aggravate postharvest mycotoxin contamination. This could imply that proactive management options are highly required. In this regard, several strategies have been proposed to effectively minimize problems associated with mycotoxigenic fungi, their toxins and loss in agricultural commodities.

These strategies are targeted to prevent fungal growth and molds on the grain substrate to reduce exposure to mycotoxins, which can be achieved by the use of synthetic fungicides [20,21], chemical inhibitors [22], drying and physical separations [23]. Most of the subsistence farmers in eastern Africa depend on synthetic fungicides. However, chemicals are blamed for increased costs, handling hazards, pesticide residues and health risks [24,25]. Thus, in an attempt to reduce the use of synthetic fungicides and due to increasing public awareness of pollutive, residual and subsequent health and environmental effects of many synthetic fungicides, the importance of alternative indigenous products to control phytopathogenic fungi is gaining popularity worldwide [26,27].

A promising alternative approach is the use of plant phytochemical constituents for inhibiting fungal growth and, hence, to control mycotoxin contamination. Previous research investigations have demonstrated the antimicrobial efficacy of several constituents of medicinal plants, herbs and weeds in the world [28], [29], [30], [31]. However, the antifungal and inhibitory potential of various medicinal, herbal and weed plants against mycotoxin producing fungi and their mycotoxins have not been well documented in Ethiopia. Therefore, the objectives of this study were to evaluate antifungal potential of medicinal, herbal and weed plant extracts against *F. verticillioides*; and to determine maximum radial growth inhibition and minimum inhibitory concentrations of effective extracts.

## 2 Materials and methods

### 2.1 Collection of medicinal, herbal and weed plants

Leaves of *Agave* sp., *Amaranthus caudatus, Anethum graveolens, Artemisia glacialis, Aloe vera, Calpurnia aurea, Cucumis prophetarum, Cymbopogon citratus, Datura stramonium, Galinsoga parviflora, Kalanchoe lanceolata, Lagenaria siceraria, Mentha* sp., *Moringa oleifera, Ocimum lamifolium, Ocimum tenuiflorum* (*sanctum*), *Olea africana, Parthenium hysterophorus, Phytolacca dodecandra, Punica granatum, Rosmerinus officinalis, Ruta chalepensis, Thymus vulgaris, Vernonia amygdalina* and *Zehneria scabra* were collected mainly from eastern Ethiopia. Collections were made during the months of June 2018 to February 2019. The test plants were selected on the basis of indigenous knowledge on their uses in traditional medicine and grain preservation. The information was obtained by approaching elders and community leaders as well as from published literatures [32,33,54]. Information on the plant species is presented in Table 1.

**Table 1:** List of medicinal, herbal and weed plant species and traditional medicinal values used in the study for their fungitoxic and fumigative potential against *Fusarium verticillioides*.

S/N	Botanical name (scientific name)	Uses/ailments treated in human beings and animals	References (cited in)
1	*Agave* sp*.*	Eye irritation, skin lesion, dandruff, indigestion & cancerous tumor (prostate and breast)	[52]
2	*Amaranthus caudatus* L.	Laxative, eye disease, amoebic dysentery & anthelminitic	[32,33]
3	*Anethum graveolens* L.	Bilharzia, heartburn, diuretic, gonorrhea, stomachache, haemorroids & digestion problem	[32,33]
4	*Artemisia glacialis*	Small pox, perfume, stomachache	[32]
5	*Aloe vera*	Earache, eye & skin disease, spleen & liver complaints, wound dressing & constipation	[32,34]
6	*Calpurnia aurea* (Ait.) Benth.	Amoebic dysentery, diarrhea, gonorrhea, hypotensive, insecticide, wound, haemorroids, eye disease, vomiting & stomachache	[32,33]
7	*Cucumis prophetarum* Jusl*. (aculeatus)*	Wound, diarrhea, migraine, snake bite, leprosy, venereal & liver diseases, rabies	[32,33]
8	*Cymbopogon citratus* (DC ex Nees) Stapf	Heart, chest & stomach complaints	[32]
9	*Datura stramonium* L. var *stramonium*	Nacrotic, headache, wound dressing, head fungus, toothache & rheumatism	[32]
10	*Galinsoga parviflora* Cavan.	Wound dressing	[32]
11	*Kalanchoe lanceolata* (Forsk.) Pers	Anthelmintic, against ascaris, diarrhea, vomiting, swelling, wound dressing & bloat	[32,33]
12	*Lagenaria siceraria*	Against ascaris, haemorroids, madness, edema & skin diseases	[33,34]
13	*Mentha* sp*.*	Condiment in tea, common cold & headache	[32]
14	*Moringa oleifera*	Possess antitumor, anti-inflammatory, antiulcer, antihypertensive, antioxidant, hepatoprotective, antibacterial & antifungal activities.	[53]
15	*Ocimum lamifolium* Hochest ex. Benth.	Cough, cold, headache, mouth blister, eye infection, fever, diarrhea & amoeba	[34,54]
16	*Ocimum tenuiflorum* (*sanctum*)	Snake & scorpion bites, eye disease, diuretic, headache, fever, cough, gonorrhea, anthelmintic & insect repellent	[33]
17	*Olea europaea* subsp. *africana* Miller	Anthelmintic, condiment in drinks & purgative	[32]
18	*Parthenium hysterophorus*	Anti-inflammatory, fever, diarrhea, malaria, dysentery, cold, neurologic disorder, urinary tract infection, herpes, skin rashes & gynecological ailments	[34,55,56]
19	*Phytolacca dodecandra* L'Herit *Phytolacca abyssinica* Hoffm. (syn.)	Amoebic dysentery, rabies, gonorrhea, eczema, jaundice, venereal disease, wound, skin disease, purgative, anthelmintic, epilepsy & haemorrhages	[32,33,54]
20	*Punica granatum* L.	Taenifuge, liver disorders, diarrhea, headache, wound dressing & anthelmintic	[32,33]
21	*Rosmerinus officinalis* L.	Condiment	[32]
22	*Ruta chalepensis* L.	Anthelmintic, epilepsy, fevers, stomach, tooth & earache, cold, heart pain, intestinal disorders, influenza & colic remedy	[32,54]
23	*Thymus vulgaris* (*Thymus* spp*.*) Hoechst ex. Benth.	Condiment, gonorrhea, cough, liver & respiratory disease	[32,54]
24	*Vernonia amygdalina* Del.	Poison antidote, tonsillitis, malaria, wound dressing, urinary inflammation, menstruation pain & skin disease	[32,33]
25	*zehneria scabra* (l.f.) sonder	diarrhea, fever, malaria, syphilis, purgative, skin diseases, anthelmintic, eye & wound dressing & headache	[32,33,54]

Identification of plant species was carried out at the Herbarium of Haramaya University, Ethiopia and based on Jansen [32], Engels et al. [33] and Singh [34]. The voucher specimens of some of the plant samples were deposited and remaining ones are to be deposited in the Herbarium of university. Once the leaf specimens were collected, samples were cleaned and dried at room temperature in dark for about 10–30 days. Some leaf samples including *Agave* sp., *A. vera, K. lanceolata* and *P. dodecandra* were cut into small pieces and dried under the same conditions. Then, air-dried leaf samples were powdered using a milling machine and the flour of each sample was kept in a cool dry place in an experimental plastic bag until ready for crude extraction [35,36].

### 2.2 Fungal culture

Fungal culture used in this study was isolated from maize grain samples collected from eastern Ethiopia. Isolation and culturing were carried out at Plant Pathology Laboratory of Haramaya University, Ethiopia. *Fusarium verticillioides*, which was isolated from mold forming maize samples, was inoculated and grown on potato dextrose agar (PDA, Difco Laboratories; Detroit, MI) and incubated in the dark at 25 °C for about 7–10 days until sporulation [37,38]. Colonies of the fungus with microscopic features of grown *F. verticillioides* were transferred to new PDA medium. The identity of the isolate was confirmed with reference cultures identified and preserved in the same laboratory. The identification of this fungus was based on cultural, morphological and description in existing publication of Raper and Fennel [39].

Before antifungal assay, *F. verticillioides* was sporulated on PDA medium for 7 days at 25 °C. Following it, spores were harvested by adding 10 ml of sterile distilled water containing 0.05 % Tween 20 and scraping the surface of the culture to free the spores. The spore suspension was further adjusted with sterile 0.05 % Tween to give a final concentration of 2 × 10^6^ spores ml^–1^ using modified procedures of El-Desouky et al. [29] and Sánchez et al. [40]. The number of spores was counted and measured using the Malassez haemocytometer slide under an optical microscope field of vision of 10x eyepiece and 40 x objectives [41].

### 2.3 Sample preparation and extraction

Fifty grams of powdered leaves of 25 test plants were soaked in 250 ml of 99.9 % methanol (v/v), stirred and thoroughly agitated using shaker and incubated at 25 ± 2 °C over night at room temperature for 24 h in the dark based on slightly modified procedures of Adiguzel et al. [42]. Next, the crude extracts were filtered first through folded Whatman No. 1 into a 500 ml round-bottom flask and subsequently evaporated to dryness, and concentrated with a rotary evaporator (Heidolph rotary evaporator Laborata 4001) at 50–55 °C and concentrated under reduced pressure to get their corresponding residues based on Lin et al. [43], Bobbarala et al. [44] and Tequida-Meneses et al. [45]. The resulting extract was placed into a desiccator until the weight was constant [37]. The total amount of crude extract yield varies per plant species. The dry residues were then dissolved in sterilized distilled water, frozen and lyophilized to get dry lyophilized power, called crude methanolic extract (CME) [36]. The yield percentage of the extract was determined using the formula suggested by Anokwuru et al. [46] as follows:

$$Yield\left(\%\right)=\frac{W2-W1}{Wo}x100$$

Where, W_2_ = the weight of the extract along with the container (flask); W_1_ = the weight of empty container; and W_o_ = the weight of the initial dried and powdered leaf sample.

### 2.4 Phytochemical screening

The presence of some phytochemical compounds was carried out on tested effective methanol crude extracts using standard procedures of Evan [47], Evan and Trease [48], Pradhan et al. [49], Yadav and Agarwala [50], and Musto et al. [51] at the Central Laboratory of Haramaya University, Ethiopia:

***Alkaloids***: 5 ml of the extract was treated first with 2 ml of HCl, and then with 1 ml of Dragendroff's reagent. Formation of an orange or red precipitate indicated the presence of alkaloids. Or 0.5 g of each sample were dissolved with 5 ml of 2 N HCl and filtered. The filtrate was treated with Dragendroff”s reagent. Formation of red precipitate indicates the presence of alkaloid.
***Flavonoids***: 1 ml of the extract was treated with few drops of 2 % NaOH to produce an intense yellow color. After adding few drops of dilute HCl, the extract became colorless if it contained flavonoids.
***Glycosides***: 1 ml of the extract was treated first with 2 ml of CH_3_COOH mixed with few drops of FeCl_3_, and then with 1 ml of H_2_SO_4_. Formation of a reddish brown color at the junction of two layers and the bluish green color in the upper layer indicated the presence of glycosides.
***Phenols***: Extract was treated with 3–4 drops of ferric chloride (FeCl_3)_ solution. Formation of bluish black color indicated the presence of phenols.
***Reducing sugars***: 1 ml of the extract was first treated with 5–8 drops of Fehling's solutions (A and B), and then heated in a water bath. Formation of a red precipitate indicated the presence of reducing sugars.
***Steroids***: 1 ml of the extracts was treated first with 10 ml of chloroform (CHCl_3_), and then with 10 ml of H_2_SO_4_. A red color in the upper layer and a yellow color in H_2_SO_4_ layer indicated the presence of steroids. Alternatively, the extract was mixed with 2 ml of CHCl_3_ and concentrated H_2_SO_4_ was added sidewise. A red color produced in the lower chloroform layer indicated the presence of steroids.
***Tannins***: 1 ml of the extract was first diluted with 4 ml of dd H_2_O, and then treated with few drops of 10 % FeCl_3_. Formation of a blue/green color indicated the presence of tannins.
***Terpenoids***: 5 ml of the extract was treated first with 2 ml of (CH_3_CO)_2_O, and then with 2 ml of CHCl_3_. Finally, 2 ml of H_2_SO_4_ were added. Formation of reddish violet color indicated the presence of terpenoids in the extract.

### 2.5 Antifungal activity assay

#### 2.5.1 Effect of phytochemicals on radial growth

Petri plates with PDA media containing 5 mg ml^–1^ of solids from crude plant extracts, which were upgraded from the preliminary study (Table 2), were centrally point inoculated with 1 × 10^6^ spores ml^–1^ from 7 days-old cultures of *F. verticillioides*. The inoculated Petri plates were incubated in darkness at 25 ± 2 °C to grow *F. verticillioides* [38]. Synthetic fungicide (benomyl) and media containing no CME were used as positive and negative controls, respectively. The radial colony diameters were measured every 24 h during the study periods with the help of a caliper. Fungal growth was measured as colony diameter and toxicity of CME against *F. verticillioides* was measured in terms of percent mycelia inhibition by the formula [36]:

$$\text{Growth}\;\text{inhibition}\;\left(\%\right)=\;\frac{\text{Dc}-\text{Dt}}{\text{Dc}}\;x\;100$$

Where, Dc = the mean value of colony diameter of the control media (mm) and Dt = the mean colony diameter of the treatment amended media (mm). The radial extension rate of the colony, *r* (mm day^–1^), was determined from the slope resulting from the radial growth versus time graph [57]. The experimental plates were arranged in completely randomized design (CRD) with three replications. The complete antifungal analysis was carried out under strict aseptic conditions [44]. The trials were repeated once.

**Table 2:** Yield of methanolic crude extracts of medicinal, herbal and weed plants (%), and antifungal activity of the extracts of against *F. verticillioides* under controlled conditions.

S/N	Botanical name	Family	Antifungal activity ^a^	Inhibition effect (0–4 scale) ^b^
1	*Agave* sp*.*	Asparagaceae	**++**	3.0
2	*A. caudatus* L*.*	Amaranthaceae	**++**	2.5
3	*A. vera*	Liliaceae	**–**	0
4	*A. graveolens* L.	Umbelliferae	**++**	3.0
5	*A. glacialis*	Asteraceae	**+++**	3.5
6	*C. aurea*	Papilionaceae	**+**	1.0
7	*C. prophetarum*	Cucurbitaceae	**+**	1.0
8	*C. citratus*	Gramineae	**+++**	3.5
9	*D. stramonium* L.	Solanaceae	**++**	2.5
10	*G. parviflora*	Asteraceae	**++**	3.0
11	*K. laceolata*	Crassulaceae	**+**	1.5
12	L. *siceraria*	Cucurbitaceae	**+**	1.0
13	*Mentha* sp*.*	Labiatae	**+**	1.5
14	*M. oleifera*	Moringaceae	**+**	1.5
15	*O. lamifolium*	Labiatae	**+**	2.0
16	*O. tenuiflorum*	Laminaceae	**+**	1.5
17	*O. europaea* subsp. *africana*	Oleaceae	**+**	1.0
18	*P. hysterophorus*	Asteraceae	**++**	2.0
19	*P. dodecandra*	Phytolaccaceae	**+**	1.5
20	*P. granatum*	Lythraceae	**+++**	3.0
21	*R. officinalis*	Labiatae	**+++**	3.0
22	*R. chalepensis*	Rutaceae	**+++**	3.5
23	*T. vulgaris*	Laminaceae	**++++**	4.0
24	*V. amygdalina*	Asteraceae	**–**	0
25	*Z. scabra*	Cucurbitaceae	**++**	2.0

Data given are mean of three replicates. ^a^ Notations were used to estimate the percentage inhibition (PI) of mycelial growth of the test fungus: – = PI = 0 %; + = PI = 11 to 30 %; ++ = PI = 31 to 50 %; +++ = PI = 51 to 70 %; ++++ = PI ≥ 71 % based on modified procedures of Thippeswamy et al. [21]. ^b^ Inhibition effect on 0–4 scale; 0 = no inhibition zone visible and 4 = inhibition zone free of fungal growth [58].

The following notations were used to estimate the percentage inhibition (PI) of mycelial growth of *F. verticillioides*: – = no antifungal activity; + = scantly antifungal activity (PI of 11 to 30 %); ++ = moderate antifungal activity (PI of 31 to 50 %); +++ = strong antifungal activity (PI of 51 to 70 %); and ++++ = very strong antifungal activity (PI ≥ 71 %) based on modified procedures of Thippeswamy et al. [21]. The degree of inhibition of fungal growth was also expressed on a 0–4 scale [58]; where, 0 = no inhibition zone visible; 1 = inhibition zone barely distinct, fungal growth and sporulation only slightly inhibited; 2 = inhibition zone well distinct, fungal growth ca. 50 % of the control, slight sporulation; 3 = inhibition zone with sparse (ca. 25 % of the control) fungal growth; and 4 = inhibition zone free of visible fungal growth.

#### 2.5.2 Effect of phytochemicals on sporulation and spore dimension

Thirty ml of potato dextrose broth (PDB) containing 150 µl of each effective extract was considered for this assay. Potato dextrose broth was prepared from fresh potato. Briefly, 200 g of fresh potato tubers were peeled and sliced into pieces, added to 1 L of water and boiled together for 40 min. Then, the broth was strained through 4 layers of cheesecloth, and the slices were discarded. The infusion (effluent) was maintained and mixed with 20 g glucose with stirring, and then enough water was added to bring the volume back to 1 L. Autoclaved, allowed to cool, and aseptically poured to Petri plates.

Spore diameter was determined according to the procedures of Harris [59]. Cover-slips were placed in Petri plates and covered with 10 ml of PDB containing the extract. Positive control contained PDB plus a volume of methanol equal to that used to dissolve the solids of the crude extract, and the negative control contained only PDB. The plates were inoculated with 20 µl of a 1 × 10^6^ spore suspension of the test fungus at 25 ± 2 °C, and its development was monitored until they germinated. One cover-slip containing spores was removed at random after 6–7 days of incubation from the plates and 100 measurements of spore diameter were carried out using micrometer under an optical microscope field of vision (10x eyepiece and 40x objective) [41]. Size measurements included both wide- and length-wise based on modified procedures of Valenzuela-Cota et al. [38]. The experimental plates were laid out in CRD with three replications. The experiment was repeated once.

### 2.6 Minimum inhibitory concentration

Since methanolic crude extracts of *T. vulgaris, C. citratus, R. officinalis, G. parviflora, R. chalepensis, Agave* sp., *A. graveolens* and *A. gracialis* exhibited strong inhibitory activity against mycelial growth, sporulation and spore size, the minimum inhibitory concentration (MIC) was determined only for these eight extracts. 1000 µl of each effective extract per 20 ml of PDA was found effective to inhibit colony radial growth of the test fungus, and this concentration was used to set the experiment for MIC. Four series of dilution including 50, 40, 30 and 10 µl ml^–1^ were prepared and considered in the MIC study of eight effective extracts. The MIC of the extracts was determined according to Szekely et al. [60].

A final concentration of 0.5 % (v/v) Tween-20 was used to enhance crude extract solubility. Potato dextrose agar medium was inoculated with 3μl aliquots of culture containing approximately 10^6^ spores ml^–1^ of the fungus of 24 h slant culture in aseptic conditions and transferred into sterile 9 inch diameter Petri dishes. The contents were incubated at 25 ± 2 °C. Petri plates without plant extracts were served as controls. Petri plates were arranged in CRD with three replicas. Inhibition of organism growth in the plates containing test crude extracts was judged by comparison with growth in blank control plates. The MICs were determined as the lowest concentration of extracts inhibiting visible growth of the fungus on the agar plate [44,61].

### 2.7 Data analysis

Data from two runs of experiments were pooled after confirming the homogeneity of variances for growth and sporulation assays using Bartlett's variance homogeneity test [62]. Descriptive statistics were used to report spore size measurements of the fungus per extract tested. Analysis of variance was performed to determine influence of methanolic crude extracts on mycelial growth of *F. verticillioides* using SAS GLM procedure [63]. Mean separations were made using the least significant difference test at 0.05 probability level. Regression analyses of colony radial growth against time after inoculation were performed and the slopes were used as measures of growth rates (mm day^–1^) in each source treatment [64].

## 3 Results

### 3.1 Preliminary screening of phytochemicals on fungal growth

**Fig. 1:**
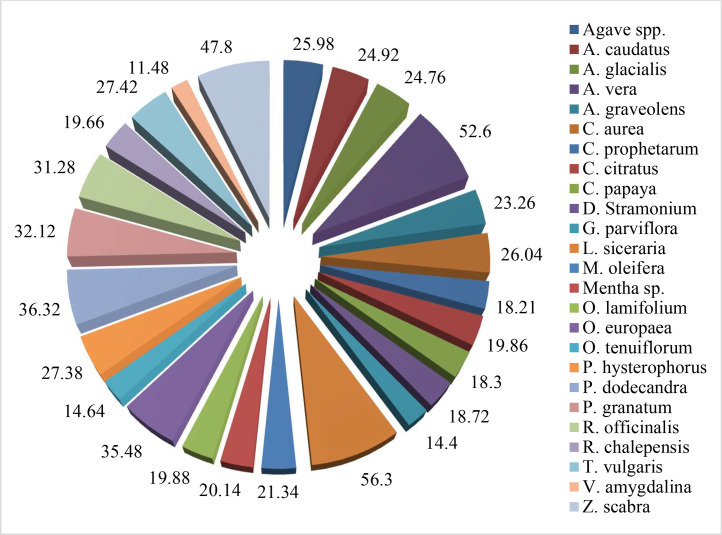
The crude yield of methanolic extracts (fresh weight basis) of medicinal, herbal and weed plants used in the study.

The yield of extracts, based on fresh weight basis, is presented in Fig. 1. The highest extract yield was obtained from L. *siceraria* (56.30% w/w), followed by *A. vera* (52.60% w/w) and *Z. scabra* (47.80% w/w). The lowest extract yield was obtained from *V. amygdalina* (11.48% w/w) and *G. parviflora* (14.40% w/w). But, variation in yield did not measure efficacy of antifungal potential of extracts. The antifungal potential of methanol extracts of medicinal, herbal and weed plants (in the preliminary study) is presented in Table 2.

Out of the 25 extracts, 23 of them showed some biological activity against the test fungus using paper disk method. The extracts exhibited variable degrees of inhibition potential against *F. verticillioides* and the effects were found to be none/weak to moderate based on the 0–4 scale. Potent activity against the test fungus was observed from *T. vulgaris*, followed by *A. glacialis, C. citratus, P. granatum, R. chalepensis* and *R. officinalis.* Intermediate antifungal potential was also exhibited by *Agave* sp., *A. caudatus, A. graveolens, D. stramonium, G. parviflora, P. hysterophorus*, and *Z. scabra*. Two extracts, namely *A. vera* and *V. amygdalina*, did not show any potency on growth of the fungus. Inhibition activity was characterized by fungal growth around the treated paper disk compared with control plates.

### 3.2 Effect of phytochemicals on radial growth

Based on the preliminary screening assay, only 13 methanolic extracts were found very potent and evaluated against different biological characteristics of the test fungus using food poisoning method. The effects of those extracts on mycelial growth are presented in Tables 3 and 4. Analysis of variance revealed that mycelia growth of *F. verticillioides* was very highly significantly (*P* < 0.0001) influenced by extracts compared with untreated control throughout the incubation period (Table 3).

**Table 3:** ANOVA for the effects of crude methanolic extracts on the mycelial growth of *F. verticillioides* during the assessment periods.

Sources of variation	df	Mean square of mycelial growth^a^					
		48	72	96	120	144	168
Extract	14	116.89^⁎⁎⁎^	361.70^⁎⁎⁎^	809.45^⁎⁎⁎^	1232.33^⁎⁎⁎^	1697.45^⁎⁎⁎^	2224.46^⁎⁎⁎^
Replication	2	3.76ns	1.82 ^ns^	0.51 ^ns^	2.52 ^ns^	3.27 ^ns^	2.42 ^ns^
Error	28	2.99	1.29	2.79	2.42	3.46	5.06
CV (%)		22.44	8.24	7.99	5.85	5.82	5.91

^a ***^ = significant at *P* < 0.0001; ns = non-significant (*P* > 0.05); and df = degrees of freedom. Mycelial growth recordings were made for six consecutive days (h) after inoculation and incubation (DAI).

**Table 4:** Antifungal potential of different plant extracts against *F. verticillioides* growth in PDA medium at effective concentration (5 mg ml^–1^) expressed as colony growth diameter (mm) at different days after inoculation and incubation at 25 ± 2 °C.

Extract/control	Colony radial growth (mm) of *F. verticillioides* measured at different days (h) after inoculation and incubation^1^						MIC (µl)^2^
	48	72	96	120	144	168	
Negative control	21.00^a^	37.00^a^	56.50^a^	66.33^a^	77.67^a^	84.00^a^	–
*A. caudatus* L*.*	16.50^b^	26.17^c^	36.33^c^	48.67^c^	56.83^c^	66.67^c^	–
*Z. scabra*	12.67^c^	28.50^b^	41.83^b^	52.67^b^	60.17^b^	72.33^b^	–
*P. hysterophorus*	12.50^c^	18.67^e^	27.83^d^	36.67^d^	46.50^d^	56.67^d^	–
*D. stramonium* L.	11.67^c^	22.17^d^	37.67^c^	48.83^c^	59.50^bc^	71.33^b^	–
*P. granatum*	11.50^c^	19.50^e^	28.17^d^	34.17^d^	40.33^e^	48.50^e^	–
*A. glacialis*	6.33^d^	12.17^f^	17.00^e^	20.00^f^	25.17^g^	30.83^g^	50
*A. graveolens* L.	5.33^de^	8.67^g^	15.67^e^	24.67^e^	33.33^f^	42.50^f^	30
*Agave* sp*.*	5.00^d–f^	7.67^gh^	10.83^f^	12.50^h^	13.33^f^	15.67^i^	40
*R. chalepensis*	4.17^d–f^	7.00^gh^	11.00^f^	12.83^gh^	15.00^i^	17.17^i^	10
*G. parviflora*	3.67^d–f^	7.17^gh^	11.00^f^	15.33^g^	18.83^h^	24.33^h^	50
*R. officinalis*	3.17^ef^	5.83^h^	10.50^f^	12.33^h^	14.67^i^	18.50^i^	40
*C. citratus*	2.17^fg^	6.50^h^	9.50^f^	13.50^gh^	18.50^h^	22.67^h^	10
*T. vulgaris*	0.00^g^	0.00^i^	0.00^g^	0.00^i^	0.00^j^	0.00 ^j^	10
Positive control	0.00^g^	0.00^i^	0.00^g^	0.00^i^	0.00^j^	0.00 ^j^	–
LSD_0.05_	2.89	1.90	2.79	2.60	3.11	3.76	
CV (%)	22.44	8.24	7.99	5.85	5.82	5.91	

Mean values in same column followed by similar letter(s) are non-significant at 5 % probability level. ^1^Radial growth was determined as average of two runs of experiments on radial colony growth of *F. verticillioides*. ^2^MIC = Minimum inhibitory concentration of eight most effective extracts noted after 120 h of incubation, – = MIC not determined for.

Throughout the study, it was found that each extract showed different effect on mycelial growth and sporulation till 7 days after inoculation (DAI) (Tables 3 and 4). However, some extracts showed weak, moderate, high and complete inhibitory effects on mycelial growth and growth rates, which clearly implied the differences in antifungal potential of extracts used. Among the extracts evaluated, *Z. scabra* showed the least (72.33 mm) mean fungistatic potential, followed by *D. stramonium* (71.33 mm), and *A. caudatus* (66.67 mm) at the final (168 h) date of growth assessment.

**Fig. 2:**
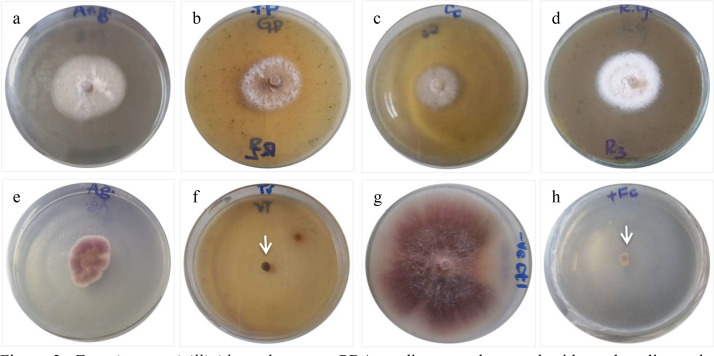
*Fusarium verticillioides* cultures on PDA medium supplemented with methanolic crude extracts of selected plants and incubated at 25 ± 2 °C for 7 days: (a) *A. gracialis*, (b) *G. parviflora*, (c) *C. citratus*, (d) *R. officinalis*, (e) *Agave* sp., (f) *T. vulgaris*, (g) negative control, and (h) positive control. The white arrows imply no radial growth due to fungistatic potential of each respective treatment.

On the contrary, *T. vulgaris* completely inhibit growth of the fungus throughout the incubation period, which was similar to the commercial benomyl fungicide used as a positive control. But, all tested extracts were effective in inhibiting any observable growth pattern in *F. verticillioides* for a 2 day period with <20 mm growth diameter. In this regard, *A. glacialis* (63.30 %), *G. parviflora* (71.04 %), *C. citratus* (73.01 %), *R. officinalis* (77.98 %), *R. chalepensis* (79.56 %), *Agave* sp. (81.35 %), and *T. vulgaris* (100 %) were the most effective extracts to control radial growth of the fungus at the tested concentration with respective reduction percentage at 7 DAI (Fig. 2). The fungus relatively showed resistance persistence to *Z. scabra, D. stramonium, A. caudatus, P. hysterophorus, P. granatum*, and *A. graveolens* with mycelial growth reduction percentages of only 13.89, 15.08, 20.63, 32.54, 42.26 and 49.40 %, respectively.

Moreover, the potent extracts caused the growth habit of the fungus to attain different patterns. In some of the extracts, the fungus had creamy, yellow, white and chocolate colony color, oozy-like mycelia growth, smooth to irregular colony shape, rough colony margin, and/or raised, light, radial, diffusive and slow to rapid mycelial growth, implying that the methanolic crude extracts not only affect radial growth but also general growth habit of the test fungus. Contrarily, typical fusarial growth, which completely covered the plate area at seven-days after inoculation and incubation (7 DAI) was observed for the negative control. Such control had radially extending light mycelia, which were regular, smooth and circular colony (Fig. 2).

Growth inhibition potentials of extracts against *F. verticillioides* were also confirmed by their potential influence on mycelial growth rates (Table 5). The highest (0.5320 mm day^–1^) growth rate of the fungus was recorded from PDA inoculated plates, followed by *D. stramonium* (0.5020 mm day^–1^), *Z. scabra* (0.4810 mm day^–1^) and *A. caudatus* (0.4230 mm day^–1^). Conversely, the lowest (0.0857 mm day^–1^) growth rate was calculated for *Agave* sp., followed by *R. chalepensis* (0.1080 mm day^–1^), *R. officinalis* (0.1250 mm day^–1^), *C. citratus* (0.1700 mm day^–1^) and *G. parviflora* (0.1701 mm day^–1^).

**Table 5:** Antifungal potential of different plant crude extracts against *F. verticillioides* growth in PDA medium at effective concentration (5 mg ml^–1^) expressed as radial growth rate (mm day^–1^) for 168 h of inoculation and incubation at 25 ± 2 °C.

Extract/control	Radial growth rate (mm day^–1^)	SE of rate a	Intercept b	SE of Intercept a	R^b^ (%) c
Negative control	0.5320	0.0245	–0.370	2.8320	96.5
*A. caudatus* L*.*	0.4230	0.0083	–3.800	0.9577	99.4
*Z. scabra*	0.4810	0.0202	–7.270	2.3300	97.1
*P. hysterophorus*	0.3730	0.0089	–7.130	1.0270	99.0
*D. stramonium* L.	0.5020	0.0092	–12.300	1.0670	99.4
*P. granatum*	0.3020	0.0068	–2.230	0.7843	99.1
*A. glacialis*	0.1960	0.0063	–2.570	0.7315	98.3
*A. graveolens* L.	0.3200	0.0236	–12.900	2.7270	91.5
*Agave* sp*.*	0.0857	0.0066	–1.580	0.7670	90.7
*R. chalepensis*	0.1080	0.0062	–0.484	0.7137	94.7
*G. parviflora*	0.1701	0.0043	–4.950	0.4975	98.9
*R. officinalis*	0.1250	0.0066	–2.670	0.7610	95.5
*C. citratus*	0.1700	0.0109	–6.180	1.2550	93.5
*T. vulgaris*	–	–	–	–	–
Positive control	–	–	–	–	–

a Standard error of parameter estimates.

b Intercept of the regression equation.

c Coefficient of determination in the model; and – sign indicates absence of fungal growth and hence, no rate value at effective concentration of a tested extract and positive control. Linear radial growth rates were estimated as the slope of the following function: Colony diameter = radial growth rate x time + b*.*

### 3.3 Phytochemical constituents

Most of the tested extracts, out of 12, in the antifungal assay contained bioactive compounds including flavonoids, steroids, terpenoids, phenols, glycosides, and tannin. Alkaloids were found only in *R. chalepensis* and *P. hysterophorus*. Moreover, *P.granatum, R. officinalis, T. vulgaris*, and *Z. scabra* were tested positive for reducing sugars. However, variable responses were observed among plant extracts to each test. Accordingly, *C. citratus, R. chalepensis*, and *T. vulgaris* (for flavonoids and steroids), *R. officinalis*, and *T. vulgaris* (for terpenoids and glycosides), *R. officinalis, Agave* sp. and *T. vulgaris* (for phenols), and *C. citratus* (for tannin) showed strong reaction for each stated bioactive phytochemical constituents (Table 6).

**Table 6:** Phytochemical constituents of methanolic crude extracts of medicinal, herbal and weed plants used in the antifungal study.

S/N	Botanical name	Alkaloid	Flavonoid	Steroids	Terpenoids	Phenols	Glycosides	Tannins	Reducing sugars
1	*Agave* sp*.*	–	+	+	+	++	+*	+*	–
2	*A. caudatus* L*.*	–	+	+	+	+	+	+	–
3	*A. graveolens* L.	–	+	+	+	+	+	+	–
4	*A. glacialis*	–	+	+	+	+	+	+	–
5	*C. citratus*	–	++	++	+	+	+	++	–
6	*G. parviflora*	–	–	+	+	+	–	+	–
7	*P. hysterophorus*	+	+	+	+	+	+	+	–
8	*P. granatum*	–	–	+	–	+	+	+	+
9	*R. officinalis*	–	+	+	++	++	++	+	+
10	*R. chalepensis*	+	++	++	+	+	+	+	–
11	*T. vulgaris*	–	+	++	++	++	++	+	+
12	*Z. scabra*	–	+	+	+	++	+	+	+

+/– = presence or absence of glycosides, tannins, phenols, alkaloids, flavonoids, saponins, steroids, terpenoids and/or reducing sugars in the effective extracts considered in the evaluation studies. ++ and * indicate extracts showing very strong and slight reaction, respectively.

### 3.4 Effect of phytochemicals on sporulation and spore dimensions

The extent of sporulation and spore dimensions were determined for each effective extract using potato dextrose broth at 7 DAI (Table 7). Broth media, which were amended with few of the extracts, continued to maintain fluid state and remained fungal growth free at the date of determination were found as a good indicator of absence of sporulation. In this regard, *Agave* sp., *C. citratus*, and *T. vulgaris* amended plates did not support sporulation. However, other extracts supported few to abundant sporulation with variable conidial dimensions. But, *Agave* sp. and *C. citratus* supported mycelial growth to some extent.

**Table 7:** Antifungal potential of different plant extracts against *F. verticillioides* growth in PDA medium at effective concentration (5 mg ml^–1^) expressed as spore size (µm) at seven days after inoculation and incubation at 25 ± 2 °C.

Extract/control	Dimensions of conidia (µm)^a^								
	Length					Width			
	Min.	Max.	Mean	SD		Min.	Max.	Mean	SD
*A. caudatus* L*.*	13.14	13.76	13.37	0.34		2.50	2.63	2.56	0.07
*A. glacialis*	18.21	18.66	18.44	0.22		3.87	3.93	3.91	0.03
*A. graveolens* L.	15.99	16.21	16.08	0.12		3.01	3.43	3.26	0.22
*Agave* sp*.*	–	–	–	–	–	–	–	–	–
*C. citratus*	–	–	–	–	–	–	–	–	–
*D. stramonium* L.	20.67	21.11	20.92	0.22		3.56	3.71	3.65	0.08
*G. parviflora*	17.34	17.78	17.62	0.24		3.11	3.21	3.15	0.05
*P. granatum*	15.04	15.75	15.34	0.37		3.21	3.32	3.28	0.06
*P. hysterophorus*	17.54	17.89	17.69	0.18		3.35	3.45	3.40	0.05
*R. chalepensis*	13.11	13.54	13.29	0.22		3.40	3.51	3.44	0.06
*R. officinalis*	15.23	15.75	15.51	0.26		3.45	3.56	3.52	0.06
*T. vulgaris*	–	–	–	–	–	–	–	–	–
*Z. scabra*	17.34	17.74	17.57	0.21		3.98	4.05	4.01	0.04
Negative control	24.16	24.65	24.34	0.27		4.10	4.22	4.18	0.07
Positive control	–	–	–	–	–	–	–	–	–

a Average of 50 readings; **–** indicated absence of sporulation and hence, no conidial dimension recorded at the effective concentration of the tested extract and positive control. SD = Standard deviation.

Tested plant extracts also showed remarkable differences in conidial dimensions. Extracts of *Agave* sp., *C. citratus*, and *T. vulgaris* did not result in sporulation and recorded none conidial dimensions, which was comparable to the positive control. Other extract treated plates recorded mean conidial length ranging from 13.37 to 20.92 µm compared with the negative control, which recorded mean conidial length of 24.34 µm. Similarly, mean conidial width of 2.56–4.01 µm was obtained from extract treated plates at effective concentration in comparison to conidial width measured from negative control plates (4.18 µm).

### 3.5 Minimum inhibitory concentration

Since the methanolic extracts of *A. glacialis, A. graveolens, Agave* sp., *C. citratus, G. parviflora, R. officinalis, R. chalepensis*, and *T. vulgaris* exhibited highly strong inhibitory activity against mycelial growth of the test fungus, the MIC was determined only for these extracts. The potato dextrose broth dilution method was used to determine the MIC (10–50 µl ml^–1^) of each extract. Extracts from *R. chalepensis, C. citratus* and *T. vulgaris* exhibited the lowest MIC value of 10 µl ml^–1^ against *F. verticillioides*. Extract from *A. graveolens* showed moderate MIC value of 30 µl ml^–1^, while crude extracts from *Agave* sp., *R. officinalis, A. glacialis*, and *G. parviflora* were lesser active than other extracts tested, which recorded MIC values ranging from 40 to 50 µl ml^–1^ (Table 4).

## 4 Discussion

Mold and mycotoxin contamination of stored maize grains is very common and a serious problem in various parts of Ethiopia [3], [4], [5]. This could be due to varied agro-climatic conditions, non-scientific methods of agricultural practices, lack of host resistance, and poor handling and storage facilities. *Fusarium verticillioides* is one of the most important phytopathogens, which causes ear rot in maize and various diseases in different crops, and is also able to produce carcinogenic fumonisins [65]. This could call for monitoring and management of such pathogens to reduce health risks and losses in agricultural commodities. But, chemical management options, which were thought to be the most effective, are prohibited due to pollutive and residual effects and subsequent health concerns [26,27].

As a result, in this study, 25 different plant extracts of medicinal, weed and herb nature were evaluated against different biological characteristics of *F. verticillioides* in response to consumers demand for safe products and health risks. Fungitoxic potential of the test extracts demonstrated that plant extracts including *A. glacialis* (63.30), *G. parviflora* (71.04 %), *C. citratus* (73.01 %), *R. officinalis* (77.98 %), *R. chalepensis* (79.56 %), *Agave* sp. (81.35 %), and *T. vulgaris* (100 %) had considerable reduction effects on radial mycelial growth of *F. verticillioides*. Similar trends were also observed on mycelial growth rates. Other related studies also documented the inhibitory potentials of phytochemicals of different origin, extraction protocols and types on several pathosystems [40,[66], [67], [68], [69], [70], [71]].

Such variability in the magnitude of potency among the extracts could be attributed to the presence and amount of different phytochemicals in the test methanolic extracts [[21], [36], [40], [57], [72], [73], [74]]. Amongst the effective methanolic extracts, *T. vulgaris* resulted in complete growth inhibition, followed by *Agave* sp., *R. chalepensis, R. officinalis, C. citratus*, and *G. parviflora*, which could be due to the presence of at least six bioactive constituents, such as steroids, terpenoids, phenols, tannins, glycosides, and flavonoids in variable proportions (Table 6). The chemical constituents are reported to have antifungal properties in which they used to demonstrate different mechanisms of inhibition to complete control of growth of the pathogenic fungi [[2], [40], [67], [75], [76], [79], [80]]. Qi et al. [71] showed that among the *T. vulgaris* essential oil, thymol (phenol) emulsion attained the strongest antifungal activity, followed by thyme oil and linalool emulsion in which the chemicals caused spore morphology changes and cell membrane destruction in *F. graminearum*. In related studies, other researchers also reported that thyme essential oils (EOs) showed better antifungal effect against *A. flavus* and *A. parasiticus* than the effects of EOs from fennel, ginger, and mint [78].

Some of the mechanisms by which phytochemicals found to inhibit mycelial growth, sporulation and spore germination in *F. verticillioides* could be contemplated to be the toxicity of the chemical constituents against the test fungus. That is, the chemical compounds which are hypothesized to localize in each phytochemical could have the ability to affect the cell membrane and cell wall integrity of the fungus, change the pH of the cellular environment in the plant tissue so that growth, sporulation and spore germination of the fungus might be hampered, alter the metabolic pathways through interfering with the functions of enzymes of the fungus, and cause structural and functional changes in cell composition. In this regard, it is reported that phenol components are toxic to microorganisms and may interfere with cell wall enzymes like chitin synthase/chitinase as well as with the α- and β-glucanases of the fungus, which would weaken cell wall integrity [75,77]. Similarly, phenolic compounds are thought to affect the permeability of cell walls and interfere with membrane functions [81].

For instance, a study by Zambonelli et al. [82] confirmed that thymol of *T. vulgaris* was correlated with cell damage, which included increase in the vacuolization of the cytoplasm and changes in the mitochondria and endoplasmic reticulum of *Colletotrichum lindemuthianum* and *Pythium ultimum*. Similarly, Qi et al. [71] found that thyme oil and thymol emulsion treatment at a concentration of 0.25 mg ml^–1^ caused morphological changes, such as severe shrinkage, rugged surface and partial membrane collapse in treated spores of *F. graminearum* only after when spores were mixed with EO emulsions and incubated for 1 h. Generally, the toxicity from phytochemical constituents could target various physiological processes in the phytopathogens, such as interfering with the synthesis of cellular walls, transport of electron, nutrient absorption, adenosine triphosphatase and other metabolic processes of the cell, altering cell permeability, deactivating various cellular enzymes and denaturing cellular proteins [83], [84], [85].

## 5 Conclusion

Due to polymorphism in bioactive compounds within the extracts, the phytochemicals strongly influenced mycelia growth, sporulation, spore germination and dimensions of *F. verticillioides* as compared to untreated control. At least six bioactive chemicals, such as steroids, terpenoids, phenols, tannins, glycosides, and flavonoids have been recovered in the test plants with variable proportions. Crude methanolic extracts from *A. glacialis, G. parviflora, C. citratus, R. officinalis, R. chalepensis*, and *Agave* sp. have had considerable reduction effects on radial mycelial growth of *F. verticillioides*, which ranged from 63.30 to 81.35 % at 7 DAI. Bioactive compounds from *T. vulgaris* found to completely inhibit mycelial growth, sporulation, and spore germination of the fungus as equivalent as the positive control (fungicide) throughout the incubation periods. Minimum inhibitory concentration of 10–50 µl ml^–1^ was recorded for the effective extracts. The overall results indicate that extracts of *T. vulgaris* could be safe source of bioactive chemicals, which are both eco-friendly and effective for the control of *F. verticillioides*. It is also a potential candidate for the development of an alternative option to synthetic fungicides to reduce the risk of maize molding under storage conditions, and it is recommended as an excellent and natural fungal control option. However, CMEs of leaves of plants were evaluated against a single fungus, which could be considered as the limitation of the study. Therefore, further studies are needed regarding choice of solvents, plants parts, concentrations, chemical fractions, modes of actions and applications, and persistence of extracts against toxigenic fungi and their mycotoxins in maize and other food grains under both in vitro and in vivo conditions.

## Data availability

All data generated are used in the article. Further datasets could be available from the corresponding author upon reasonable request.

## CRediT authorship contribution statement

**Habtamu Terefe:** Conceptualization, Data curation, Visualization, Writing – original draft, Writing – review & editing. **Getnet Yitayih:** Data curation, Visualization, Writing – review & editing. **Getachew G. Mengesha:** Data curation, Visualization, Writing – review & editing.

## Declaration of Competing Interest

The authors declare that they have no known competing financial or personal interests that could have appeared to influence the work reported in this manuscript.

## Data Availability

Data will be made available on request.
